# Sending a Signal: Evaluating the Impact of Program and Geographic Preference Signaling on the Internal Medicine Residency Match

**DOI:** 10.7759/cureus.67379

**Published:** 2024-08-21

**Authors:** Dustin L Norton, Karl M Richardson, William C Lippert, Jessica Valente, Donna M Williams

**Affiliations:** 1 Pulmonary and Critical Care Medicine, Wake Forest University School of Medicine, Winston-Salem, USA; 2 Cardiology, Wake Forest University School of Medicine, Winston-Salem, USA; 3 Internal Medicine, Wake Forest University School of Medicine, Winston-Salem, USA

**Keywords:** holistic review residency application, application inflation, internal medicine residency, supplemental application, geographic preference, electronic residency application services (eras), national residency match program (nrmp) match, residency match

## Abstract

Background

For over a decade, the number of residency applications has surged, a trend known as "application inflation." COVID-19 further intensified this trend, leading the Association of American Medical Colleges (AAMC) to address the issue by introducing a supplemental application in the 2021-2022 cycle, allowing programs to identify applicants with a connection to their program or geographic region. For the 2022-2023 cycle, the number of program signals increased from five to seven. The impact of the supplemental application and the increase in signals on the likelihood of an applicant matching with a program has yet to be evaluated.

Methods

This retrospective cohort study evaluated the impact of program signaling and geographic preference on the matching likelihood in our internal medicine residency program. Data from MyERAS® and the Supplemental Application for 640 applicants who applied to our large, urban, university-based program in the Southeastern United States during the 2020-2021 and 2022-2023 application cycles were included. Using univariate and multivariate analysis, we examined the correlation between program signal, geographic preference, and final match location.

Results

Applicants who sent a program signal had nearly three-fold higher odds of matching with our program. Geographic preference was numerically but not statistically associated with higher odds of matching. Both signaling a preference for matching with a program in an urban environment and couples matching correlated with decreased odds of matching with our program. Geography was an important predictor of match location as residing in our AAMC geographic region, our four-state area, and our specific state had increased odds of matching with our program.

Conclusions

Signaling our program was associated with increased odds of matching with our program. Geographic preferences were less predictive of a match with our program; however, they did predict the likelihood of a match at a program within that region. Future studies are needed to ensure external validity.

## Introduction

For more than a decade, senior medical students have been applying to an increasing number of residency programs, a phenomenon known as “application inflation” [1]. Despite match rates for senior United States allopathic medical students remaining relatively constant over this period, the average length of rank-order lists for these applicants nearly doubled from 2003 to 2021 [2]. Internal medicine residency applications have mirrored these trends, and this phenomenon was further compounded by the COVID-19 pandemic, likely due to several factors, including a switch to virtual interviews, which decreased the cost of applying broadly, as well as a pause on away rotations [3]. From the 2020 to 2021 cycles, there was a 6% increase in the number of applicants for internal medicine residency positions with each applicant applying to an average of seven more programs compared to the previous year [4]. Many factors have contributed to application inflation [5], but unfortunately, no intervention to date has eased this pressure, leading to an increased burden on applicants and programs alike [6].

Adding applicants’ preferences early in the match process was shown to be a potential tool in this application arms race [7], and for the 2021 application cycle, the Association of American Medical Colleges (AAMC) added an optional supplemental application through the Electronic Residency Application Service® (ERAS®) for internal medicine. The supplemental ERAS application consisted of three sections which allowed applicants to convey geographic and program-specific preferences and detail impactful past experiences. Program signals enabled applicants to identify up to five programs for which they had a specific preference at the time of application. Geographic preferences allowed each applicant to identify up to three geographic regions in which they preferred to match for residency. Geographic regions are defined by the AAMC and include 13 mutually exclusive regions throughout the United States and Puerto Rico. Additionally, applicants could specify a preference for urban or rural settings. Each question of signal in the supplemental application was optional. The supplemental application aimed to help applicants highlight their interests in a program/area amidst the growing number of applications and to help programs identify a smaller, more focused pool of applicants. This approach was intended to promote a more holistic application review process [8].

After the successful debut of the supplemental application in 2021, program and geographic preference signals were incorporated into the ERAS application in 2022. Also, the number of program signals increased from five to seven. Notably, data regarding the impact of the supplemental application has been limited. While program and geographic signaling were recently shown to increase the odds of a student receiving an interview and obtaining a match position [9,10], the impact of the supplemental application on the likelihood of matching with a specific program and how the increase from five to seven program signals has affected this impact has yet to be evaluated.

Our primary objective was to evaluate the impact of program signaling and geographic preference on the likelihood of a candidate matching with our internal medicine residency program.

## Materials and methods

Our internal medicine residency program is a large, university-based program located in an urban center in the Southeastern United States, recruiting 34 residents each year. This retrospective cohort study used manually imported data from the MyERAS® curriculum vitae and the Supplemental Application. The sample included all interviewed applicants on our rank list for the 2022 and 2023 match seasons who were ranked at or above our lowest-ranked matched applicant, representing those who would have matched with our program had they chosen to.

For each applicant in the cohort, we collected these variables using a standardized case report form: the state of the applicant’s hometown, undergraduate institution, and medical school; age; sex; under-represented in medicine status; couples matching status; United States Medical Licensing Examination (USMLE) Step 2 Clinical Knowledge (CK) score; program signal; geographic preference; and urban/rural preference. Urban/rural preference was made dichotomous as urban preference versus other. Seeking a better understanding of the impact of geography on the eventual match location, we identified a four-state area that included our state and the three bordering states. In addition to documenting if the applicant had ever resided (hometown, undergraduate institution, or medical school) in this four-state area, we also recorded whether an applicant had ever resided in our much larger AAMC-defined region or our state. We defined program affinity as applicants having any identifiable draw to our region or our program (e.g., sending a program signal, providing a geographic preference, or ever having lived or trained in our AAMC-defined geographic region). The chosen variables were determined by our study team based on subject content knowledge and drawing from data points that would be quickly available for pre-interview selection and review of applications.

In our application review process, program signals and geographic preferences prioritized applications for earlier review. Once invited for an interview, these signals were no longer used to determine an applicant’s position on our rank list, and interviewers were not provided information regarding an applicant’s program signal, geographic preference, or urban/rural preference. No changes were made to the interview process between the 2021-2022 and 2022-2023 interview cycles. We hypothesized that program signals, geographic preferences, and current and past applicant geography would predict the likelihood that a ranked candidate would match our program. Chi-square tests were used for dichotomous variables, and t-tests were used for continuous variables to compare those who matched in our program versus elsewhere. Multivariable logistic regression models evaluated independent match predictors, with covariates selected based on significance in univariate comparison or at the discretion of the investigators. IBM SPSS Statistics for Windows, Version 27 (Released 2020; IBM Corp., Armonk, New York, United States) was used for data analysis, and p < 0.05 was the statistical significance threshold used.

## Results

About 640 applicants were eligible for analysis, of whom 67 matched with our program. Table 1 displays our cohort and univariate comparisons between those who matched with us and those who did not. Of the matched applicants, 60 (89.6%) preferred our AAMC-defined region, 40 (59.7%) had previously resided in our AAMC-defined region, and 35 (52.2%) sent a signal to our program. The percentage of matched applicants who signaled our program increased numerically from 2022 to the 2023 match (42.4% (14/33) versus 61.8% (21/34), p = 0.113), though this finding was not statistically significant. In univariate analysis, sending a program signal was associated with an increased frequency of matching with our program when compared with not sending a program signal (35/189 (18.5%) versus 32/451 (7.1%), p < 0.001). In a multivariable model controlling for sex, couples match, USMLE Step 2 CK score, urban preference, geographic preference, and prior geography, a program signal continued to be strongly associated with increased odds of matching with our program (OR 2.62, 95% CI 1.54-4.46, p < 0.001). There was minimal numerical difference in the strength of this association from the 2022 to 2023 match (OR 2.45 versus 2.56).

**Table 1:** Cohort demographics and applicant data SD: standard deviation; USMLE Step 2 CK: United States Medical Licensing Examination Step 2 Clinical Knowledge Examination; URiM: underrepresented in medicine; four-state region: North Carolina, Virginia, Tennessee, South Carolina; AAMC region: South Atlantic AAMC region

Applicant characteristics	Match at our program (n = 67)	Match elsewhere (n = 573)	p-value
Age, years (SD)	26.79 (2.40)	26.95 (2.13)	0.57
Female gender, n (%)	29 (43.3)	289 (50.4)	0.27
USMLE Step 2 CK, score (SD)	252.7 (8.8)	255.1 (9.8)	0.054
Couples match, n (%)	3 (4.5)	76 (13.3)	0.039
URiM, n (%)	6 (9.0)	75 (13.1)	0.59
Urban preference, n (%)	30 (44.8)	351 (61.3)	0.034
Geographic preferences, n (%)			
Preference our region	60 (89.6)	442 (77.1)	0.019
Preference our region or no geographic preference	63 (94.0)	524 (91.4)	0.47
Historical geography, n (%)			
Ever resided in our state	10 (14.9)	115 (20.1)	0.32
Ever resided in four-state region	23 (34.3)	221 (38.6)	0.50
Ever resided in AAMC region	40 (59.7)	332 (57.9)	0.78
Program signal	35 (52.2)	154 (26.9)	<0.001

Using the same full cohort of 640 applicants in the same multivariable model, signaling a geographic preference of our AAMC-defined South Atlantic region was numerically, but not statistically, associated with an increase in the odds of matching with our program after controlling for our covariates (OR 2.13, 95% CI 0.92-4.94, p = 0.078). Having resided in our AAMC-defined region was similarly not associated with matching in our program. Couples matching correlated with decreased odds of matching with our program (OR 0.29, 95% CI 0.09-0.97, p = 0.036), while signaling a preference for matching in an urban environment also trended toward decreased odds of matching with our program, despite the AAMC classifying our program as urban (OR 0.63, 95% CI 0.39-1.01, p = 0.055). When evaluating only those applicants who did not send a signal to our program (n = 451), sending a geographic preference signal for our region did not significantly increase the odds of matching with our program (OR 1.60, 95% CI 0.59-4.40, p = 0.36). Among this same cohort of applicants who did not send us a program signal, and in the same multivariable model, living in our AAMC region was not related to matching at our program (OR 1.41, 95% CI 0.65-3.04, p = 0.39), nor was living in our four-state region (OR 1.02, 95% CI 0.48-2.18).

**Figure 1:**
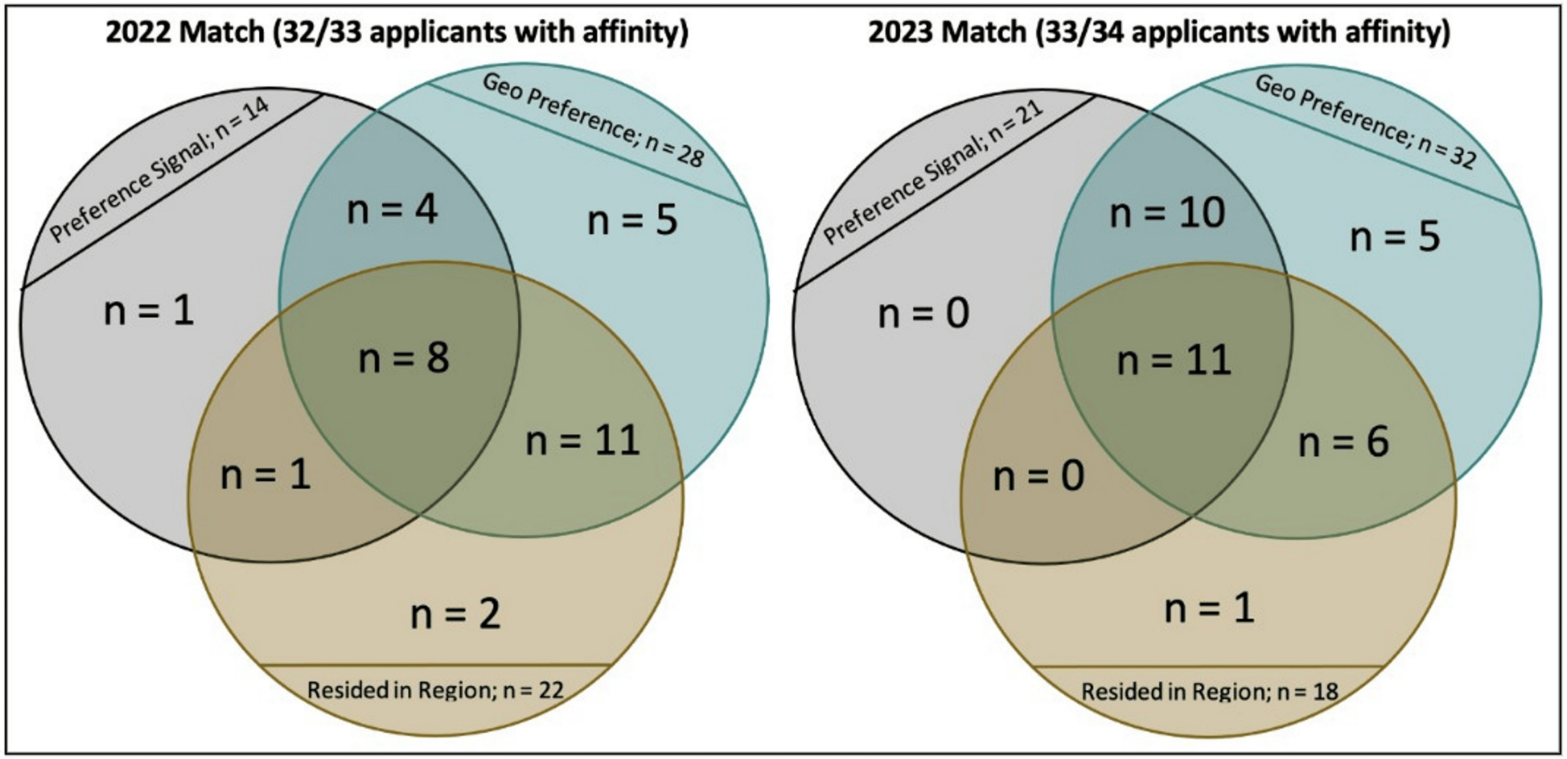
Visualizing affinity for our program and region in 2022 and 2023 match

Of all applicants who matched with our program over the two years, only 3% (2/67) had no markers of the program or regional affinity (i.e., did not signal our program, did not preference our AAMC region, or had not previously resided (hometown or prior training) within our AAMC region). In our multivariable model, having any of these markers of program or regional affinity increased the odds of matching at our program by over four times (OR 4.53, 95% CI 1.06-19.29, p = 0.041) compared to having none of these. Figure 1 compares this affinity for our program and region among matched applicants between the 2022 and 2023 matches.

Regarding all applicants included in our study and their ultimate match location, geography does appear to be an important predictor of geographic match location in general, if not to a specific program. In a similar multivariable model including all applicants, preferencing our AAMC-defined geographic region was associated with matching in the same region (OR 1.60, 95% CI 1.05-2.43, p = 0.030). This geographic region preference was not associated with matching in our four-state area or our state; however, having resided in our AAMC geographic region was the strongest predictor of matching in that region (OR 3.05, 95% CI 2.15-4.32, p < 0.001). Similarly, residing in our four-state area (North Carolina, South Carolina, Virginia, Tennessee) was a strong predictor of matching within our four-state area (OR 1.98, 95% 1.40-2.80, p < 0.001), as was living in our state a predictor of matching in our state (OR 1.70, 95% CI 1.07-2.71, p = 0.024). Urban preference was associated with decreased odds of matching in our four-state area (p = 0.049) and our state (p = 0.008), but not our AAMC-defined region. Table 2 shares a summary of the results of our multivariable modeling.

**Table 2:** Results of multivariable modeling AAMC: American Association of Medical Colleges; AAMC region: South Atlantic AAMC region; four-state area: North Carolina, Virginia, Tennessee, South Carolina

Dependent variable	Covariates	Significant or relevant predictors	Odds ratio (95% CI)	p-value
Program match (n = 640)	Sex, couples match, urban preference, geographic preference, prior region geography, program signal	Program signal	2.62 (1.54-4.46)	<0.001
		Couples match	0.29 (0.09-0.97)	0.036
		Urban preference	0.63 (0.39-1.01)	0.055
		Geographic preference	2.13 (0.92-4.94)	0.078
		Prior AAMC region geography	0.98 (0.57-1.69)	0.95
Program match, applicants who did not signal (n = 451)	Sex, couples match, urban preference, geographic preference, prior region geography	Geographic preference	1.60 (0.59-4.40)	0.39
		Prior AAMC region geography	1.41 (0.65-3.04)	0.39
Program match (n = 640)	Sex, couples match, urban preference, any marker of program/regional affinity	Program affinity	4.53 (1.06-19.29)	0.041
		Urban preference	0.56 (0.36-0.88)	0.013
AAMC region match (n = 640)	Sex, couples match, urban preference, geographic preference, prior region geography	Geographic preference	1.60 (1.05-2.43)	0.030
		Prior AAMC region geography	3.05 (2.15-4.32)	<0.001
		Urban preference	0.78 (0.57-1.06)	0.11
		Couples match	0.72 (0.43-1.18)	0.19
Four-state area match (n = 640)	Sex, couples match, urban preference, geographic preference, prior four-state geography	Geographic preference	1.39 (0.90-2.15)	0.14
		Prior four-state geography	1.98 (1.40-2.80)	<0.001
		Urban preference	0.73 (0.53-0.999)	0.049
		Couples match	0.95 (0.56-1.60)	0.83
Our state match (n = 640)	Sex, couples match, urban preference, geographic preference, prior state geography	Geographic preference	1.70 (0.98-2.95)	0.059
		Prior state geography	1.70 (1.07-2.71)	0.024
		Urban preference	0.61 (0.43-0.88)	0.008
		Couples match	0.43 (0.20-0.93)	0.031

## Discussion

Inflation in residency applications has inflicted increasing pressure on applicants and programs alike [1, 4-6]. Program signaling and geographic preferences were introduced to help manage this inflation and ease these pressures, allowing applicants an opportunity to highlight unique aspects of their application and signal specific interests they may have in a program or region [7]. For residency programs, the supplemental applications helped to identify an enriched pool of applicants to allow and encourage a more holistic review of applications [8]. Importantly, for these signals to decrease the pressures of application inflation, it is vitally important that the assumption that these applicants are more likely to match with a particular program must be verified. After the 2022 match, we proposed to assess our assumption that program signals and geographic preferences would be associated with the likelihood that an applicant would match our program. We expanded on this by using information from the 2023 match to reassess this assumption and to evaluate the impact of an increased number of program signals. Our data support these assumptions, and our study lays a foundational framework that can be used by other programs to identify factors that correlate with the likelihood of matching with their specific program.

In this retrospective study of the 2021-2022 and 2022-2023 application cycles, program signaling was the strongest predictor of matching with our program (OR 2.62, p < 0.001). Of the 67 matched residents included in our analysis, 52% sent a signal to our program. These results are similar to what was found in a survey conducted by Szumel et al. among senior US allopathic medical students in North Carolina, which found that 47% sent a program signal to the program with which they ultimately matched [11]. Notably, this survey was limited by a relatively low response rate (with only 39 out of 85 contacted students completing the supplemental application and related questions), whereas our study benefits from having information on a larger number of applicants, which helps reduce selection bias. 

Geographic preference alone was not as strong of a predictor as we had expected (OR 2.13, p = 0.078), and this association was even weaker when used, as was the case for application review by our program after program signals were evaluated first and removed from the cohort (OR 1.60, p = 0.36). These results are also consistent with findings from Szumel et al. which found that applicants were 2.95 times as likely to receive an interview invitation from programs if they had sent a program signal but only 1.75 times as likely to receive an interview from a program in an indicated geographic preference region [11]. Applicants having any identifiable affinity to our region or our program, meaning sending a program signal, having a geographic preference, or ever having lived or trained in our AAMC-defined geographic region, were much more likely to match with our program when compared to applicants with none of these signals (OR 4.53, p = 0.041). Of 67 matched applicants, only two (3%) did not have at least one of these identifiable affinities.

Overall, >50% of applicants who matched with our program had signaled our program. Although this percentage increased from 42% to 62% from 2022 to 2023, this change was not statistically significant. This lack of significance may be due to insufficient power and the overall increase in the number of signals, resulting in more signals in both the matched and unmatched groups of applicants. Nonetheless, applicants that signaled our program had odds 2.6 times greater of matching with our program, and this association remained similar despite the increase in signals allowed (2.45 versus 2.56).

In terms of geographic area and the likelihood an applicant would match with our program, it has been noted anecdotally that the AAMC-defined regions are often exceptionally large and heterogeneous. For example, our residency program is in the AAMC South Atlantic region, which stretches along the eastern seaboard from Delaware to Puerto Rico and includes both swaths of rural southern farmland and large metropolitan areas such as the District of Columbia, Atlanta, and Miami. Therefore, we evaluated if a geographic preference for our AAMC region would correlate with a higher likelihood of matching with our program, and also if having ever lived or trained in proximity to our state might be more impactful. While only marginally significant, applicants who showed preference for our AAMC region had 2.1 times higher odds of matching with our program. Pragmatically, geographic preference is most valuable to a program in the cohort of applicants who did not send a program signal. Surprisingly, in these 451 applicants, a geographic preference of our area was less impactful (OR 1.60, p = 0.36). This lack of statistical association may speak to the lack of specificity provided by geographic preference regions.

Our findings are similar to what was reported in Benjamin et al.’s 2024 study of 970 categorical and preliminary internal medicine residents using the Texas Seeking Transparency in Application to Residency (STAR) survey [8]. They found that a program signal alone (without a geographic preference) increased the odds of matching by 4.8 times (OR 4.8, p < 0.01). Similarly, the geographic preference alone was not as strong of a predictor but still increased the overall odds of matching with a particular program by 1.7 times (OR 1.7, p < 0.01).

Interestingly, despite our program technically residing within an urban area, urban preference and couples matching tended to decrease the odds of matching with our program. Similar trends were noted when reviewing predictors of matching anywhere within our state and region. We postulated that the inverse relationship with urban preference may have been related to fewer large urban centers in the area. The inverse relationship with couples matching was at first unexpected but could be explained by considering the logistics of the couples match. Applicants may prefer cities or regions with a greater density of medicine programs to increase their likelihood of matching at geographically proximal programs.

As the number of applications to our residency program continues to rise, this information in future application cycles may help identify groups of applicants who are more likely to match our program, thus helping to alleviate the time and costs associated with the interview process. Given the strong value of program signaling and the apparent lesser value of geographic preference, increasing the number of program signals and narrowing the scope of geographic preferencing may improve the value of this information for programs and further enhance an applicant’s ability to genuinely signal interest. 

Our study has limitations. Our cohort represents a relatively small sample size. Our data is limited to a single, large, university-based internal medicine residency program; generalizing our results to other programs may be challenging. Further, our cohort contained some selection bias because we were more likely to interview applicants who signaled and geographically preferred our region. This inherently associates our covariates with our outcome to a degree. Notably, our results remained very consistent across our two years of data despite an increase in the number of program signals available, increasing the internal validity of our results. Future multicenter studies are needed to better evaluate these associations to determine if there is external validity to our results. Likely the strength of these associations will vary from one program to another, though results within each institution may be helpful in determining the best process for each individual program to approach application review. 

It should be noted that preliminary analyses from the 2021-2022 application cycle were previously presented in abstract form at the 2023 Alliance of Academic Internal Medicine Spring meeting in Austin, Texas.

## Conclusions

Program signaling appears to be a valuable tool for residency programs to identify applicants who have a higher likelihood of matching with their program. A program signal to our residency program correlated with nearly three times higher odds of matching with our program. Geography, both signaled preference and lived preference, appears to be less strongly correlated with program match, though it does tend to predict the final region that an applicant will match in. Though these are unlikely to have external validity, other programs can use their own internal results to find variables that may correlate with a likelihood of matching their program. Future studies are needed to improve the ability of programs to interview candidates most likely to choose their program as a match.
